# Drug survival of ixekizumab, TNF inhibitors, and other IL‐17 inhibitors in real‐world patients with psoriasis: The Corrona Psoriasis Registry

**DOI:** 10.1111/dth.14808

**Published:** 2021-02-15

**Authors:** Benjamin Lockshin, Angel Cronin, Ryan W. Harrison, Robert R. McLean, Laura Anatale‐Tardiff, Russel Burge, Baojin Zhu, William N. Malatestinic, Bilal Atiya, Mwangi J. Murage, Gaia Gallo, Bruce Strober, Abby Van Voorhees

**Affiliations:** ^1^ US Dermatology Partners Rockville Maryland USA; ^2^ Corrona LLC Waltham Massachusetts USA; ^3^ Eli Lilly and Company Indianapolis Indiana USA; ^4^ Yale University New Haven Connecticut USA; ^5^ Central Connecticut Dermatology Research Cromwell Connecticut USA; ^6^ Eastern Virginia Medical School Norfolk Virginia USA

**Keywords:** biologics, ixekizumab, psoriasis, registries, tumor necrosis factor inhibitors

## Abstract

To compare drug survival of ixekizumab to other IL‐17 inhibitors (IL‐17i) and TNF inhibitors (TNFi) among patients with psoriasis (PsO) in a real‐world setting. Participants included adult PsO patients enrolled in the Corrona Psoriasis Registry who initiated ixekizumab, TNFi, or other IL‐17i between 16 March 2016 to 10 August 2019 and completed ≥1 follow‐up visit. Multivariable adjusted hazard ratios (HR) were calculated to estimate the risk for drug discontinuation in the ixekizumab group relative to the other drugs. Among the 1604 drug initiations, 552 initiated ixekizumab, 450 initiated TNFi, and 602 initiated other IL‐17i. Mean age was 51 years, 49% were women, and 52% were obese (BMI > 30). Ixekizumab patients had a higher proportion of patients with PASI >12 at drug initiation (24%) than TNFi (15%) and other IL‐17i (19%). Over a median of 11 months of follow‐up, 723/1604 (45%) drug discontinuations occurred. Persistence of ixekizumab, TNFi, and other IL‐17i at 24‐months were 68%, 33%, and 46%, among biologic‐naïve patients (n = 543), and 46%, 23%, and 36%, for biologic‐experienced patients (n = 1061), respectively. Ixekizumab patients had a 64% lower risk of discontinuation vs TNFi (HR = 0.36; 95% CI 0.27‐0.47) and a 31% lower risk vs other IL‐17i (HR = 0.69, 95% CI 0.55‐0.87) after adjustment for biologic experience and other covariates. HRs were similar when limited to patients with moderate‐to‐severe PsO (BSA > 3, PASI > 3, and IGA > 1, n = 1076) at initiation. In our study of real‐world patients with PsO, initiators of ixekizumab had more prolonged drug survival than both initiators of TNFi and other IL‐17i up to 2 years of follow‐up.

## 1 INTRODUCTION

Psoriasis negatively impacts quality of life and work productivity,^1^, ^2^, ^3^ and is associated with several other comorbidities, including cardiovascular disease, Crohn's disease, depression, and anxiety.^4^ Traditional therapies for PsO include topical corticosteroids, retinoids, ultraviolet B (UVB), narrowband UVB, and systemic therapies such as methotrexate and ciclosporin. The development of highly effective biologic treatments such as IL‐17 antagonists and IL‐12/23 antagonists has transformed the treatment of PsO,^5^ offering dermatologists several therapeutic options for their patients with moderate‐to‐severe PsO in addition to the well‐known tumor necrosis factor (TNF)‐α inhibitors. Choice of optimal treatment for PsO can depend on several factors, and it is strongly driven by a drug's efficacy and safety profile. Randomized clinical trials (RCT) are still considered the best source of data when assessing such features, but they are mostly designed to address regulatory purposes under ideal and controlled circumstances in a selected patient population. The real‐life setting is a much more complex environment, where the actual drug utilization can be biased by many factors depending on the treatment strategy adopted by the clinician, the drug, and the patient in a specific health care system. Therefore, describing the performance of any particular treatment in real‐life is a more appropriate tactic to measure effectiveness. Drug survival has been extensively considered as a surrogate of drug effectiveness in real life. It is the duration of time a patient remains on the same drug. It can be impacted by several factors such as drug factors (ie, efficacy and safety profile, tolerability), health care system factors and access to the therapy, and factors related to the patient preference and adherence to the clinician prescription.^6^

Ixekizumab, a high binding affinity monoclonal antibody that selectively targets IL‐17A, approved for the treatment of moderate‐to‐severe PsO in adults, has been recently approved for moderate‐to‐severe pediatric PsO patients (ages 6 to under 18). In rheumatology, ixekizumab has been approved for active psoriatic arthritis, ankylosing spondylitis, and nonradiographic axial spondyloarthritis (nr‐axSpA).^7^ Ixekizumab has demonstrated superior efficacy to placebo, etanercept, ustekinumab, and guselkumab in several large, randomized head‐to‐head clinical trials.^8^, ^9^, ^10^ Furthermore, the safety profile of ixekizumab has demonstrated to be significantly consistent with up to 5 years of continuous treatment and across all PsO trials.^11^, ^12^, ^13^, ^14^, ^15^, ^16^, ^17^, ^18^, ^19^, ^20^ In a recent study of long‐term safety data in 13 ixekizumab clinical trials, Genovese et al.^21^ found that when compared across 17 331.1 patient years (PY) of exposure in PsO (n = 5898 patients with ≥4 years of exposure), rates for adverse events (AEs) remained largely stable or declined.

When investigating select categories of adverse events (AEs) (ie, serious infections, opportunistic infections), inflammatory bowel disease (IBD) (eg, Crohn's disease, ulcerative colitis, and indeterminate IBD), and serious hypersensitivity events among all patients exposed to ixekizumab in the Corrona PsO Registry, Grace et al^22^ determined the exposure‐adjusted incidence rates (IRs) of safety events did not increase with increasing time of ixekizumab exposure. Moreover, the IRs of safety outcomes during the first 24 months of treatment among ixekizumab‐treated patients were consistent with those observed in PsO clinical trials,^23^, ^24^ and no new safety signals were identified.

Currently, there are few studies comparing the drug survival for ixekizumab to that of other biologic therapies in real‐world patients with PsO. Two recent claims‐based studies of US patients demonstrated greater drug survival for ixekizumab vs adalimumab^3^ and secukinumab patients.^25^ In addition to the demonstrated favorable ixekizumab drug survival compared with these specific treatments (adalimumab and secukinumab), there is a need to understand whether similar findings are observed when ixekizumab users are compared with other biologics‐users, overall, among PsO patients. Whether ixekizumab has longer persistence needs confirmation from different data sources.

The objective of the current study was to compare drug survival of ixekizumab to that of TNF inhibitors (TNFi) and non‐ixekizumab IL‐17 inhibitors (IL‐17i) among real‐world patients with PsO in the Corrona Psoriasis Registry.

## 2 METHODS

### 2.1 Study setting

This analysis used data from the Corrona Psoriasis Registry, a prospective, multicenter observational disease‐based registry launched in April 2015 in collaboration with the National Psoriasis Foundation, the design of which has been previously described.^26^ As of October 2019, Corrona had enrolled 9519 patients and had data on 26 739 patient visits. Patients were recruited from 229 private and academic practice sites, with 469 participating dermatologists, in the United States and Canada across 45 states and provinces. Patients were enrolled if they met the following criteria: 18 years or older, PsO diagnosed by a dermatologist, and initiated or switched to a US Food and Drug Administration (FDA) approved systemic or biologic PsO treatment at registry enrollment or within 12 months before enrollment. Data were collected using questionnaires completed by patients and their treating dermatologists at regular office visits at approximately 6‐month intervals.

### 2.2 Study population

The study population included 1604 patient drug initiations (ixekizumab, n = 552; TNFi [adalimumab, certolizumab, etanercept], n = 450; and non‐ixekizumab IL‐17i [secukinumab, brodalumab], n = 602) that occurred between 22 March 2016 (FDA approval date for use of ixekizumab for plaque PsO) and 10 August 2019 (the data cut for the current study analysis). Initiations were required to occur at or after registry enrollment, initiators had at least one subsequent follow‐up (FU) visit, and an available index visit (defined as the visit coinciding with or within 4 months before initiation) at which baseline characteristics were assessed.

### 2.3 Outcome

The primary outcome was drug survival, defined as persistent use of drug during follow‐up, allowing for a medication gap of ≤60 days. Drug survival time was defined from the date of drug initiation through the date of drug discontinuation. Patients persistent on drug at their last follow‐up visit were censored on the date of their last follow‐up visit.

### 2.4 Covariates

Baseline characteristics included: sociodemographics (eg, age, gender, race, education, insurance, work status, geographic region, smoking, weight, body mass index [BMI], history of comorbidities); PsO disease characteristics (eg, erythrodermic morphology, inverse/intertriginous morphology, nail morphology, disease duration, comorbid psoriatic arthritis); disease severity (eg, Psoriasis Area and Severity Index (PASI), body surface area (BSA), Investigator Global Assessment (IGA); prior systemic therapy (biologic and nonbiologic); concomitant PsO treatment (topical therapy); and patient‐reported outcomes (PROs) (eg, patient‐reported overall itch/pruritis, skin pain, fatigue; patient‐reported health state today [EQ‐VAS]; Dermatology Life Quality Index [DLQI]; and Work Productivity and Activity Impairment [WPAI] questionnaire).

### 2.5 Statistical analysis

Baseline characteristics were summarized descriptively for the treatment groups (ixekizumab, TNFi, non‐ixekizumab IL‐17i). Pairwise standardized differences were used to assess differences in characteristics between the treatment groups. A standardized difference effect size is equivalent to a *Z* score of a standard normal distribution and a threshold greater than 0.1 was the criteria used to represent a relevant imbalance between groups. Additionally, thresholds of 0.2, 0.5, and 0.8 are often used to indicate small, medium, and large effect sizes.^27^ Kaplan‐Meier (KM) methods were used to estimate the probability of drug survival over time for each treatment group, separately for biologic‐naïve and biologic‐experienced patients. Cox proportional hazard models, with a shared frailty term assumed to follow a gamma distribution to account for within‐patient correlation were used to estimate adjusted hazard ratios (HR) for drug discontinuation in the ixekizumab group relative to the TNFi and non‐ixekizumab IL‐17i groups.^28^, ^29^ Hazard ratios of less than 1 indicate a lower risk of discontinuation for the ixekizumab‐treated group compared with those treated by other biologics, which corresponds to prolonged drug survival among the ixekizumab‐treated group. Model 1 (base model) was adjusted for the prespecified covariates of age, gender, weight, comorbid psoriatic arthritis, duration of psoriatic disease, and history of prior biologic therapy (Yes/No?). Model 2 was adjusted for those in the base model plus select patient demographic and clinical characteristics that differed between the three treatment groups in Table 1, as evidenced by (*d*) > 0.10. Selection of variables was based upon examination of collinearity between candidate variables (ie, base model variables plus those with [*d*] > 0.10), to avoid multicollinearity in Model 2. The additional covariates selected for adjustment in Model 2 were race, education, work status, geographic region, smoking history, history of Crohn's disease, ulcerative colitis, indeterminate IBD, and other gastrointestinal (GI) disorders, history of infections, erythrodermic morphology, inverse/intertriginous morphology, nail morphology, IGA, PASI, concomitant topical therapy, patient‐reported overall itch/pruritis, and patient‐reported health state today (EQ‐VAS).

**TABLE 1:** Patient sociodemographics, comorbidities, disease characteristics, and disease activities at index visit for patients who initiated ixekizumab, TNFi (adalimumab, certolizumab, etanercept), or non‐ixekizumab IL‐17i (secukinumab, brodalumab) at or after enrollment

Total (N)	IXE	TNFi	Non‐IXE IL‐17i	Standardized difference	
				IXE vs TNFi	IXE vs non‐IXE IL‐17i
	N = 552	N = 450	N = 602		
Sociodemographics					
Age in years, Mean (SD)	50.0 (13.6)	49.3 (13.9)	51.7 (14.0)	0.05	0.13
Gender, Female, n (%)	253 (45.8)	226 (50.2)	299 (49.7)	0.09	0.08
Race, n (%)				0.11	0.02
White, n (%)	410 (74.3)	334 (74.2)	453 (75.2)		
African‐American, n (%)	22 (4.0)	17 (3.8)	23 (3.8)		
Asian, n (%)	65 (11.8)	42 (9.3)	67 (11.1)		
Other, n (%)	55 (10.0)	57 (12.7)	59 (9.8)		
Education, n (%)	n = 552	n = 450	n = 601	0.14	0.17
12th grade or less, n (%)	33 (6.0)	40 (8.9)	54 (9.0)		
High school graduate/GED, n (%)	128 (23.2)	112 (24.9)	166 (27.6)		
Some college/associates degree, n (%)	168 (30.4)	140 (31.1)	167 (27.8)		
College graduate or higher, n (%)	223 (40.4)	158 (35.1)	214 (35.6)		
Work status, n (%)	n = 551	n = 450	n = 600	0.26	0.22
Full‐time, n (%)	350 (63.5)	238 (52.9)	325 (54.2)		
Part‐time, n (%)	42 (7.6)	46 (10.2)	45 (7.5)		
Work at home, n (%)	30 (5.4)	38 (8.4)	54 (9.0)		
Student, n (%)	7 (1.3)	12 (2.7)	13 (2.2)		
Disabled, n (%)	40 (7.3)	49 (10.9)	50 (8.3)		
Retired, n (%)	82 (14.9)	67 (14.9)	113 (18.8)		
Geographic region, n (%)	n = 551	n = 450	n = 602	0.23	0.12
US Northeast, n (%)	142 (25.8)	92 (20.4)	145 (24.1)		
US Midwest, n (%)	96 (17.4)	53 (11.8)	103 (17.1)		
US South, n (%)	208 (37.7)	204 (45.3)	259 (43.0)		
US West, n (%)	85 (15.4)	79 (17.6)	79 (13.1)		
Canada, n (%)	20 (3.6)	22 (4.9)	16 (2.7)		
Smoking history, n (%)	n = 548	n = 445	n = 598	0.16	0.10
Never smoked, n (%)	265 (48.4)	229 (51.5)	292 (48.8)		
Former smoker, n (%)	193 (35.2)	125 (28.1)	189 (31.6)		
Current smoker, n (%)	90 (16.4)	91 (20.4)	117 (19.6)		
Body weight in kilograms, n	n = 550	n = 449	n = 601		
Mean (SD)	95.9 (25.0)	91.1 (25.1)	90.7 (23.6)	0.19	0.22
Body mass index (BMI)	n = 548	n = 446	n = 599		
BMI, >30 kg/m^2^ (obese), n (%)	319 (57.8)	224 (49.8)	298 (49.5)	0.16	0.17
History of comorbidities^a^					
Hypertension, n (%)	224 (40.6)	168 (37.3)	245 (40.7)	0.07	0.00
Diabetes mellitus, n (%)	90 (16.3)	80 (17.8)	117 (19.4)	0.04	0.08
Crohn's disease, n (%)	2 (0.4)	10 (2.2)	2 (0.3)	0.17	0.01
Ulcerative colitis, n (%)	1 (0.2)	6 (1.3)	2 (0.3)	0.13	0.03
Indeterminate IBD and other GI disorders, n (%)	65 (11.8)	69 (15.3)	95 (15.8)	0.10	0.12
Infections, n (%)	244 (44.2)	175 (38.9)	268 (44.5)	0.11	0.01
Disease characteristics					
Psoriasis morphology^a^					
Erythrodermic, n (%)	19 (3.4)	7 (1.6)	16 (2.7)	0.12	0.05
Inverse/intertriginous, n (%)	52 (9.4)	38 (8.4)	38 (6.3)	0.03	0.12
Scalp, n (%)	222 (40.2)	171 (38.0)	225 (37.4)	0.05	0.06
Nail, n (%)	116 (21.0)	63 (14.0)	100 (16.6)	0.19	0.11
Duration of psoriasis disease in years	n = 546	n = 450	n = 602		
Mean (SD)	16.7 (12.8)	12.2 (12.3)	16.0 (13.7)	0.36	0.05
<5 years, n (%)	98 (18.0)	166 (36.9)	147 (24.4)	0.45	0.18
5 to <10 years, n (%)	88 (16.1)	73 (16.2)	84 (14.0)		
10 to <15 years, n (%)	98 (18.0)	58 (12.9)	93 (15.4)		
15 to <20 years, n (%)	65 (11.9)	39 (8.7)	82 (13.6)		
≥20 years, n (%)	197 (36.0)	114 (25.3)	196 (62.6)		
Psoriatic arthritis—dermatologist identified, n (%)	258 (46.7)	230 (51.1)	327 (54.3)	0.09	0.15
Psoriatic arthritis—rheumatologist confirmed	n = 325	n = 306	n = 437		
n (%)	71 (21.8)	46 (15.0)	79 (18.1)	0.18	0.09
Duration of psoriatic arthritis disease in years, n	n = 258	n = 230	n = 327		
Mean (SD)	8.8 (9.7)	5.3 (7.7)	7.5 (9.0)	0.40	0.14
Disease activity					
BSA	n = 551	n = 449	n = 602	0.09	0.11
Mild disease (0, 3), n (%)	84 (15.2)	64 (14.3)	108 (17.9)		
Moderate disease (3, 10), n (%)	251 (45.6)	209 (46.5)	263 (43.7)		
Severe disease (10, 20), n (%)	98 (17.8)	68 (15.1)	91 (15.1)		
Very severe disease (20, 100), n (%)	118 (21.4)	108 (24.1)	140 (23.3)		
PASI (score: 0‐72), n					
Mean (SD)	8.9 (8.2)	7.3 (6.5)	7.5 (7.2)	0.22	0.19
PASI > 12, n (%)	130 (23.6)	67 (14.9)	115 (19.1)	0.22	0.11
IGA	n = 552	n = 450	n = 601	0.19	0.22
0: clear, n (%)	10 (1.8)	18 (4.0)	35 (5.8)		
1: almost clear, n (%)	25 (4.5)	19 (4.2)	31 (5.2)		
2: mild, n (%)	91 (16.5)	69 (15.3)	101 (16.8)		
3: moderate, n (%)	310 (56.2)	272 (60.4)	329 (54.7)		
4: severe, n (%)	116 (21.0)	72 (16.0)	105 (17.5)		

*Note*: Standardized differences >0.10 are suggestive of an imbalance between groups and thresholds of 0.2, 0.5, and 0.8 are often used to indicate small, medium, and large effect sizes (Cohen, 1988); IXE, ixekizumab; TNFi (adalimumab, certolizumab, etanercept); non‐ixekizumab IL‐17i (non‐IXE) (secukinumab, brodalumab).

Abbreviations: BMI, body mass index; BSA, body surface area; PASI, Psoriasis Area and Severity Index; IGA, Investigator Global Assessment.

a Not mutually exclusive.

Sensitivity analyses were conducted for the subset of initiations with moderate‐to‐severe disease activity at baseline (BSA > 3, PASI > 3, and IGA > 1). The proportional hazards assumption was evaluated for the models by examining Schoenfeld residuals^30^ and was met for drug group. However, there was evidence to suggest deviation from proportional hazards for some covariates (eg, weight, history of infection). Sensitivity analyses were conducted using time‐dependent coefficients for such covariates, which is one option for handling nonproportional hazards.^31^

## 3 RESULTS

**FIGURE 1:**
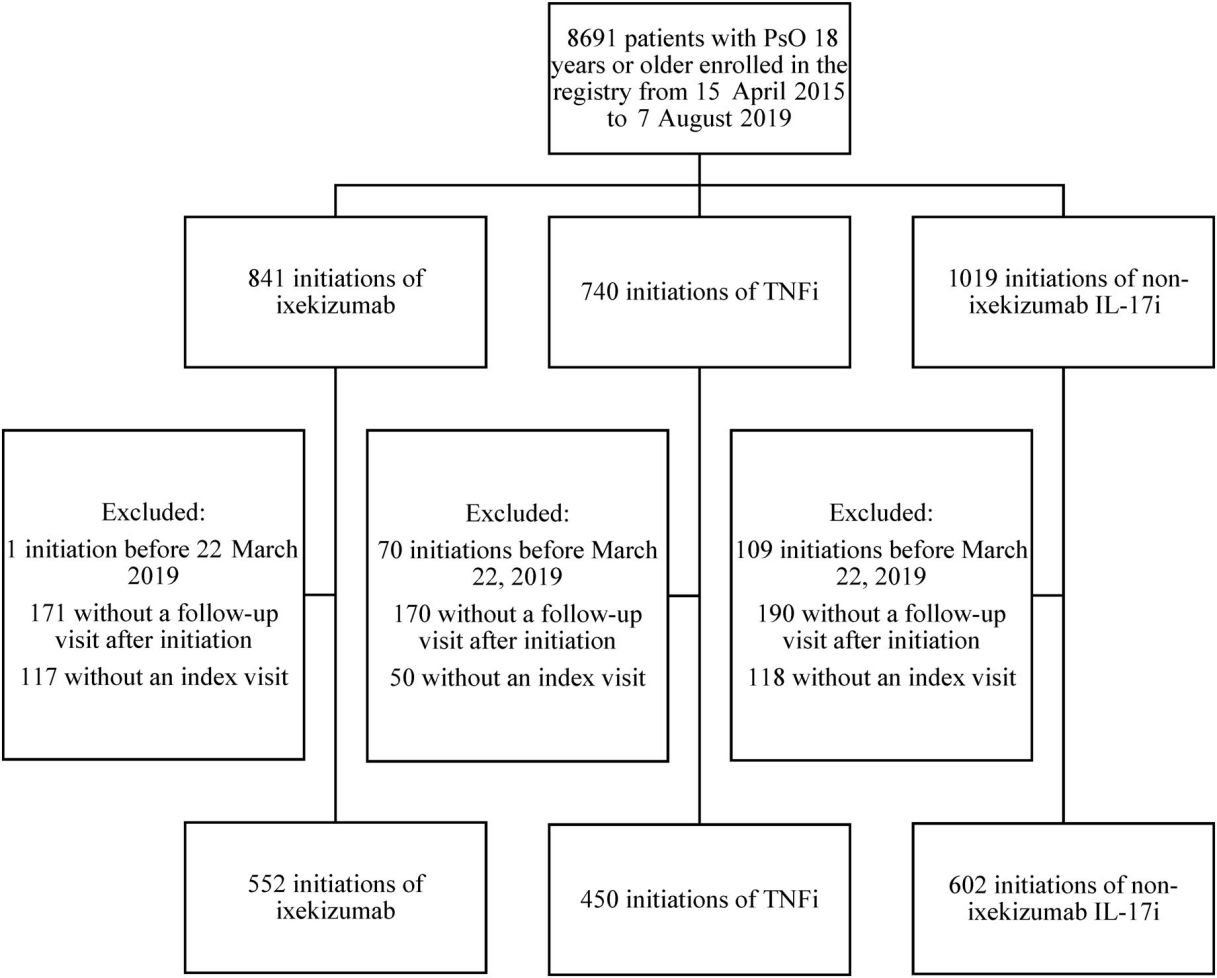
Patient attrition flow chart

There were 1604 initiations among 1412 unique patients that met the inclusion criteria for analysis of drug survival (Figure 1). Of the initiations, 34% (n = 543) occurred among biologic‐experienced patients, while 66% (n = 1061) occurred among biologic‐naïve patients. Frequencies of initiations among the three drug groups were 34% (n = 552) for ixekizumab, 28% (n = 450) for TNFi, and 38% (n = 602) for non‐ixekizumab IL‐17i. Within the biologic‐naïve subgroup, most initiations were TNFi (53% TNFi, 21% ixekizumab, and 27% non‐ixekizumab IL‐17i). In contrast, within the biologic‐experienced subgroup, the fewest initiations were TNFi (16% TNFi, 41% ixekizumab, and 43% non‐ixekizumab IL‐17i).

Patient sociodemographics and comorbidities at index visit by drug group are summarized descriptively in Table 1. For all patients, the mean age was 50.5 years, 49% were women, 75% were white, and 52% were obese (BMI > 30). In comparison to initiators of TNFi and non‐ixekizumab IL‐17i, ixekizumab initiators were more likely to work full‐time (64% ixekizumab, 53% TNFi, and 54% non‐ixekizumab IL‐17i) and be obese (BMI > 30) (58% ixekizumab, 50% TNFi, and 50% non‐ixekizumab IL‐17i).

In comparison to initiators of TNFi and non‐ixekizumab IL‐17i, initiators of ixekizumab had a higher prevalence of nail PsO (21% ixekizumab, 14% TNFi, and 17% non‐ixekizumab IL‐17i). Duration of PsO was comparable between initiators of ixekizumab and non‐ixekizumab IL‐17i (median, 13 years for both groups), while duration of disease for initiators of TNFi was shorter (median, 8 years). Comorbid psoriatic arthritis was reported by dermatologists for 47% of initiators of ixekizumab, which was slightly lower than that reported for initiators of TNFi (51%), and non‐ixekizumab IL‐17i (54%). Compared with TNFi and non‐ixekizumab IL‐17i initiators, ixekizumab initiators had a higher proportion of patients with PASI >12 (24% ixekizumab, 15% TNFi, and 19% non‐ixekizumab IL‐17i) and severe IGA (IGA = 4 on a 4‐point scale) (21% ixekizumab, 16% TNFi, and 18% non‐ixekizumab IL‐17i) (Table 1).

Table 2 presents patient‐reported outcomes (PROs) and treatment histories at index visit, by drug group. For PROs, there were meaningful differences among the three drug groups for DLQI (ixekizumab vs TNFI, *d* = 0.12; ixekizumab vs non‐ixekizumab IL‐17i, *d* = 0.15), but not for patient global assessment of PsO (ixekizumab vs TNFI, *d* = 0.03; ixekizumab vs non‐ixekizumab IL‐17i, *d* = 0.04). When evaluating WPAI domains, TNFi initiators had a higher mean overall percent of work hours affected by psoriasis (18.9 vs16.1) compared with ixekizumab initiators, although the effect size was small (*d* = 0.12). Ixekizumab initiators reported lower mean patient‐reported overall fatigue (VAS range 0‐100) (36.6 ixekizumab, 42.7 TNFi, and 39.5 non‐ixekizumab IL‐17i) and more ixekizumab initiators (53%) had taken ≥2 previous biologics than patients on TNFi (17%) and non‐ixekizumab IL‐17i (40%).

**TABLE 2:** Patient‐reported outcomes (PROs) and treatment history at index visit for patients who initiated ixekizumab, TNFi (adalimumab, certolizumab, etanercept), or non‐ixekizumab IL‐17i (secukinumab, brodalumab) at or after enrollment

Total (N)	IXE N = 552	TNFi N = 450	Non‐IXE IL‐17i N = 602	Standardized difference	
				IXE vs TNFi	IXE vs non‐IXE IL‐17i
Patient‐reported outcomes (PROs)					
DLQI effect on life	n = 552	n = 449	n = 602	0.12	0.15
0–1: none, n (%)	64 (11.6)	55 (12.2)	94 (15.6)		
2‐5: small, n (%)	172 (31.2)	131 (29.2)	180 (29.9)		
6‐10: moderate, n (%)	146 (26.4)	128 (28.5)	149 (24.8)		
11–20: very large, n (%)	141 (25.5)	121 (26.9)	159 (26.4)		
21‐30: extremely large	29 (5.3)	14 (3.1)	20 (3.3)		
WPAI					
Currently employed, n (%)	395 (71.6)	281 (62.4)	377 (62.6)	0.19	0.19
Absenteeism, n	n = 357	n = 252	n = 337		
Mean (SD)	3.8 (14.0)	5.0 (16.0)	3.4 (11.7)	0.08	0.03
Presenteeism, n	n = 353	n = 250	n = 335		
Mean (SD)	14.6 (23.0)	16.7 (22.4)	15.0 (21.3)	0.09	0.02
Work productivity loss, n	n = 352	n = 248	n = 334		
Mean (SD)	16.1 (24.5)	18.9 (24.1)	16.4 (22.7)	0.12	0.01
Activity impairment, n	n = 549	n = 445	n = 597		
Mean (SD)	23.8 (29.0)	25.7 (28.2)	23.6 (27.3)	0.07	0.01
Patient global assessment of psoriasis	n = 551	n = 450	n = 601		
Mean (SD)	49.3 (29.5)	50.1 (28.4)	48.0 (29.4)	0.03	0.04
Patient overall itch/pruritis (VAS range 0‐100), n	n = 551	n = 449	n = 602		
Mean (SD)	52.8 (33.1)	53.5 (33.2)	49.3 (33.6)	0.02	0.11
Patient overall fatigue (VAS range 0‐100), n	n = 551	n = 448	n = 600		
Mean (SD)	36.6 (30.1)	42.7 (30.8)	39.5 (30.0)	0.20	0.10
Patient overall skin pain (VAS range 0–100), n	n = 551	n = 449	n = 601		
Mean (SD)	34.7 (32.1)	34.2 (31.8)	32.5 (31.9)	0.01	0.07
Patient‐reported health state today (EQ‐VAS range 0‐100), n	n = 550	n = 444	n = 599		
Mean (SD)	70.5 (21.5)	67.7 (21.7)	70.0 (20.7)	0.13	0.02
Treatment characteristics					
Concomitant therapies^a^					
Nonbiologic systemics, n (%)	61 (11.1)	51 (11.3)	68 (11.3)	0.01	0.01
Phototherapy, n (%)	14 (2.5)	17 (3.8)	17 (2.8)	0.07	0.02
Topical agents, n (%)	249 (45.1)	239 (53.1)	268 (44.5)	0.16	0.01
History of biologic therapy				1.02	0.28
0 previous biologic agents received, n (%)	114 (20.7)	285 (63.3)	144 (23.9)		
1 previous biologic agent received, n (%)	144 (26.1)	88 (19.6)	219 (36.4)		
≥2 previous biologic agents received, n (%)	294 (53.3)	77 (17.1)	239 (39.7)		
History of nonbiologic systemic therapy				0.08	0.08
0 previous nonbiologic systemic agents received, n (%)	252 (45.7)	198 (44.0)	250 (41.5)		
1 previous nonbiologic systemic agent received, n (%)	203 (36.8)	182 (40.4)	240 (39.9)		
≥2 previous nonbiologic systemic agents received, n (%)	97 (17.6)	70 (15.6)	112 (18.6)		

*Note*: Standardized differences >0.10 are suggestive of an imbalance between groups and thresholds of 0.2, 0.5, and 0.8 are often used to indicate small, medium, and large effect sizes (Cohen, 1988).

Abbreviations: IXE, ixekizumab; DLQI, Dermatology Life Quality Index; TNFi (adalimumab, certolizumab, etanercept); non‐ixekizumab IL‐17i (non‐IXE) (secukinumab, brodalumab); VAS, visual analogue scale; WPAI, Work Productivity and Activity Impairment Questionnaire (Absenteeism = % work hours missed, Presenteeism = % impairment while working, Work productivity loss = % impairment while working, and Activity impairment = % daily activities affected by PsO); EQ‐5D‐3L (EuroQol) consists of 2 pages: the EQ‐5D descriptive system and the EQ visual analogue scale (EQ‐VAS).

^a^ Not mutually exclusive.

**FIGURE 2:**
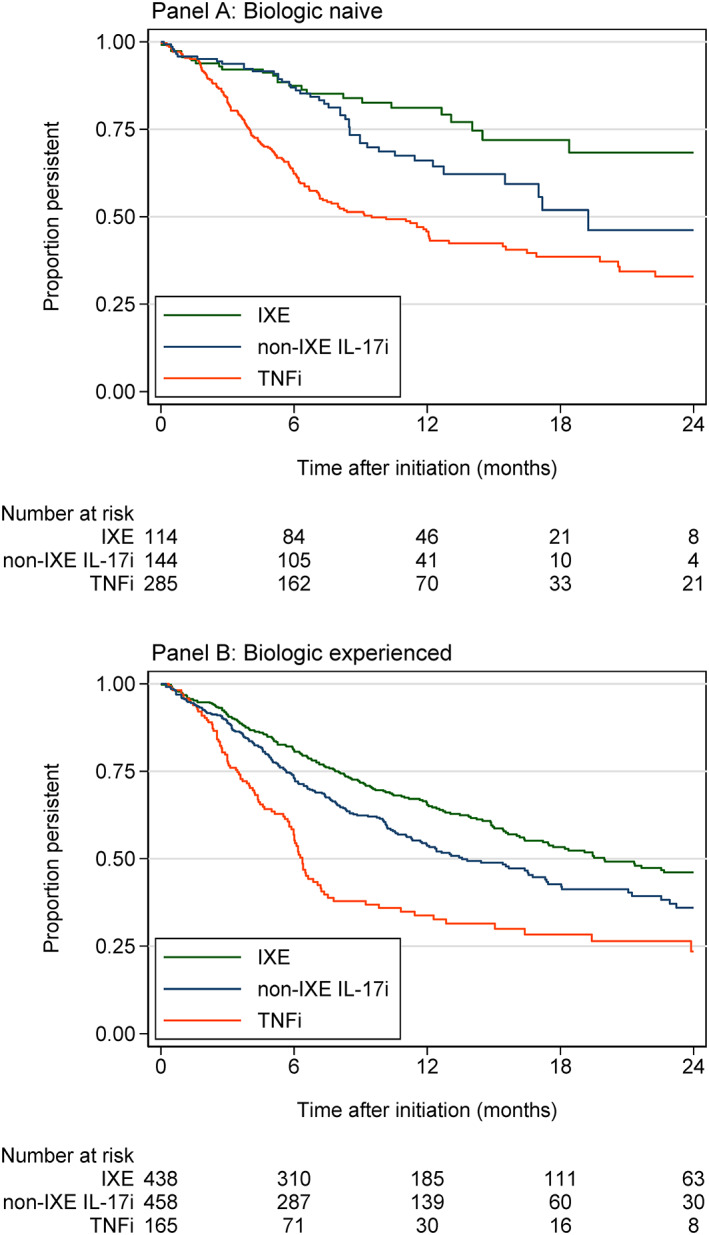
A,B, Kaplan–Meier estimates of drug survival following initiation of ixekizumab (IXE), TNFi, and non‐ixekizumab IL‐17i (non‐IXE‐IL‐17i) for biologic‐naïve and biologic‐experienced patients

There were 723 drug discontinuations among all patient initiations. The median follow‐up time for those persistent at their last Corrona visit was 11 months (interquartile range [IQR] 6‐17 months; range 1‐38 months), and for all initiations, including those resulting in discontinuation, was 8 months (IQR 5‐14 months, range 1‐38 months). Among biologic‐naïve patients, ixekizumab showed a drug survival trajectory that started diverging versus the non‐ixekizumab IL‐17i after the first 6 months of treatment, at which point ixekizumab began to demonstrate increased persistency out to 24 months (Figure 2). Drug survival at 12 months following initiation was 81% (95% confidence interval [CI], 72%‐88%) for ixekizumab, and 66% (95% CI, 56%‐74%) for non‐ixekizumab IL‐17i; respective estimates at 24 months following initiation were 68% (95% CI, 54%‐79%), and 46% (95% CI, 30%‐61%) (Table 3). The TNFi group had the lowest drug survival, with 46% (95% CI, 39%‐52%) and 33% (95% CI, 25%‐41%) at 12 and 24 months, respectively. Among biologic‐experienced patients, similar patterns were observed (Figure 2B), though persistency overall was lower: survival at 12 months was 65% (95% CI, 60%‐70%) for ixekizumab, 34% (95% CI, 26%‐42%) for TNFi, and 54% (95% CI, 48%‐59%) for non‐ixekizumab IL‐17i; respective estimates at 24 months following initiation were 46% (95% CI, 40%‐42%), 23% (95% CI, 15%‐33%), and 36% (95% CI, 29%‐43%) (Table 3).

**TABLE 3:** Kaplan‐Meier estimates of drug survival at 12‐months and 24‐months following initiation of ixekizumab, TNFi, and non‐ixekizumab IL‐17i, within biologic‐naïve and biologic‐experienced subgroups

	Main analysis^a^		Sensitivity analysis^b^ (subgroup with BSA > 3, PASI > 3, and IGA > 1 at index)	
	12‐month drug survival (95% CI)	24‐month drug survival (95% CI)	12‐month drug survival (95% CI)	24‐month drug survival (95% CI)
Biologic‐naïve				
Ixekizumab	81% (72%–88%)	68% (54%–79%)	83% (72%‐90%)	71% (56%‐82%)
TNFi	46% (39%‐52%)	33% (25%‐41%)	50% (42%‐57%)	33% (24%‐43%)
Non‐ixekizumab IL‐17i	66% (56%‐74%)	46% (30%‐61%)	66% (54%‐76%)	43% (25%‐59%)
Biologic‐experienced				
Ixekizumab	65% (60%‐70%)	46% (40%‐42%)	67% (61%‐73%)	45% (38%‐53%)
TNFi	34% (26%‐42%)	23% (15%‐33%)	33% (23%‐44%)	24% (14%‐35%)
Non‐ixekizumab IL‐17i	54% (48%‐59%)	36% (29%‐43%)	55% (49%‐61%)	38% (30%–45%)

Abbreviations: BSA, Body surface area; CI, confidence interval; IGA, Investigator Global Assessment; PASI, Psoriasis Area and Severity Index.

^a^ In the main analysis, there were 543 biologic‐naïve initiations (ixekizumab, n = 114; TNFi, n = 285; non‐ixekizumab IL‐17i, n = 144) and 1061 biologic‐experienced initiations (ixekizumab, n = 438; TNFi, n = 165; non‐ixekizumab IL‐17i, n = 458).

^b^ In the sensitivity analysis, there were 393 biologic‐naïve initiations (ixekizumab, n = 94; TNFi, n = 201; non‐ixekizumab IL‐17i, n = 98) and 683 biologic‐experienced initiations (ixekizumab, n = 296; TNFi, n = 105; non‐ixekizumab IL‐17i, n = 282).

In unadjusted Cox regression analyses, the ixekizumab group had a 67% lower relative risk of discontinuation in comparison to the TNFi group (hazard ratio [HR] = 0.33; 95% CI, 0.25‐0.43, *P* < .001), and a 34% lower relative risk of discontinuation in comparison to the non‐ixekizumab IL‐17i group (HR = 0.66, 95% CI, 0.51‐0.84, *P* < .001). Hazard ratios were like the observed data after adjustment in multivariable models. The adjusted hazard ratio from Model 2 for the ixekizumab group in comparison to the TNFi group was 0.36 (95% CI, 0.27‐0.47, *P* < .001), and for the ixekizumab group in comparison to other non‐ixekizumab IL‐17i group was 0.69 (95% CI, 0.55‐0.87, *P* = .002) (Table 4, Tables S1 and S2).

**TABLE 4:** Unadjusted and adjusted hazard ratios for the difference in drug survival for initiators of ixekizumab vs initiators of TNFi and non‐ixekizumab IL‐17i, based on Cox proportional hazards regression models

	Main analysis			Sensitivity analysis (subgroup with BSA > 3, PASI > 3, and IGA > 1 at index)		
	HR	95% CI	*P* value	HR	95% CI	*P* value
Unadjusted						
Drug group	n = 1604 initiations n = 723 discontinuations			n = 1076 initiations n = 483 discontinuations		
Ixekizumab vs TNFi (ref)	0.33	0.25, 0.43	<.001	0.34	0.25, 0.45	<.001
Ixekizumab vs non‐ixekizumab IL‐17i (ref)	0.66	0.51, 0.84	<.001	0.61	0.46, 0.81	<.001
Adjusted model 1 (base model^a^)						
Drug group	n = 1594 initiations n = 722 discontinuations			n = 1070 initiations n = 483 discontinuations		
Ixekizumab vs TNFi (ref)	0.33	0.25, 0.43	<.001	0.31	0.23, 0.43	<.001
Ixekizumab vs non‐ixekizumab IL‐17i (ref)	0.68	0.54, 0.86	.001	0.61	0.46, 0.80	<.001
Adjusted model 2 (base model plus consideration of covariates with standardized differences >0.10^b^)						
Drug group	n = 1566 initiations n = 710 discontinuations			n = 1051 initiations n = 472 discontinuations		
Ixekizumab vs. TNFi (ref)	0.36	0.27, 0.47	<.001	0.35	0.26, 0.47	<.001
Ixekizumab vs. non‐ixekizumab IL‐17i (ref)	0.69	0.55, 0.87	.002	0.62	0.48, 0.82	<.001

*Note*: Hazard ratios (HR) <1 correspond to prolonged drug survival.

Abbreviations: BSA, body surface area; CI, confidence interval; IGA, Investigator Global Assessment; PASI, Psoriasis Area and Severity Index.

^a^ Adjusted for prespecified covariates of age, gender, weight, comorbid psoriatic arthritis, duration of psoriatic disease, and history of prior biologic therapy.

^b^ Adjusted for those in model 1 plus race, education, work status, geographic region, smoking history, history of Crohn's disease/ulcerative colitis/indeterminate IBD/other GI disorders, history of infections, erythrodermic morphology, inverse/intertriginous morphology, nail morphology, PASI, IGA, concomitant topical therapy, patient‐reported overall itch/pruritis, and patient‐reported health state today (EQ‐VAS).

In sensitivity analyses, estimates of drug survival and hazard ratios were similar within the subgroup of patients with moderate‐to‐severe disease activity at baseline (Tables 3 and 4). Additionally, regression results were robust to sensitivity analyses using time‐dependent coefficients for covariates where the proportional hazards assumption may not have been met: the adjusted hazard ratio (Model 2) for the ixekizumab group in comparison to the TNFi group was 0.36 (95% CI, 0.27‐0.47, *P* < .001), and for the ixekizumab group in comparison to the non‐ixekizumab IL‐17i group was 0.69 (95% CI, 0.55‐0.87, *P* < .002).

## 4 DISCUSSION

Among patients with PsO in a large North American registry, ixekizumab demonstrated higher drug survival compared with TNFi and non‐ixekizumab IL‐17i therapies. Patterns of drug survival by drug group over 24‐months of follow‐up were similar in both biologic‐naïve and biologic‐experienced patients. Although ixekizumab initiators compared with TNFi and non‐ixekizumab IL‐17 initiators were more likely biologic‐experienced, obese, had a higher prevalence of nail morphology, worked full‐time, and had more severe disease, drug survival differences favorable to ixekizumab initiators remained even after adjusting for potential confounders. These factors, in particular previous biologic experience, often have a negative impact on drug survival. However, in our study, ixekizumab still showed better drug survival.

Drug survival of TNFi such as adalimumab^3^ and etanercept^17^, ^32^, ^33^, ^34^, ^35^, ^36^, ^37^ and a newer non‐ixekizumab IL‐17i such as secukinumab^25^ have been reported. Comparison of the results from this study and other prior studies is difficult as drug survival rates differed and decreased over time among biologic therapies. According to a meta‐analysis of 37 studies with 32 631 patients, drug survival of all biologics decreased with time. At year one, drug survival for etanercept was 66% but declined to 41% at year four, for adalimumab the drug survival rate decreased from 69% to 47%, and for ustekinumab 82% to 56%, respectively.^17^ However, high statistical heterogeneity was found within the meta‐analysis as the authors were not able to adjust for baseline patient characteristics such as age, gender, body weight, comorbidities, and co‐treatments for PsO, all factors which may influence drug survival. Differences between our estimates of drug survival and those in this study are most likely due to differences in baseline characteristics, as well as natural trends over time, since the meta‐analysis encompassed studies published as early as 2010.

Because line of therapy influences drug survival, analyses were stratified by biologic‐naïve and biologic‐experienced patients. In the current study, 66% (n = 1061) occurred among biologic‐naïve patients, and 34% (n = 543) of initiations occurred among biologic‐experienced patients. Although TNFi are still largely adopted as a first line biologic, among our biologic‐naïve patients, the TNFi group had the lowest drug survival, with 46% and 33% at 12 and 24 months, respectively. As expected, drug survival was worse among biologic‐experienced patients compared with biologic‐naïve patients, yet in both cohorts, ixekizumab had better survival than TNFi and non‐ixekizumab IL‐17i. Our 12‐month drug‐survival rates among biologic‐experienced patients of 65% for ixekizumab vs 34% for TNFi, and 54% for non‐ixekizumab IL‐17i initiators, are comparable to those recently reported for adalimumab (12 months survival: 55% for ixekizumab vs 47% for adalimumab)^3^ and secukinumab (12 months survival: 57% for ixekizumab vs 50% for secukinumab).^25^ The higher drug survival observed among ixekizumab users is consistent despite most users being biologic‐experienced, a subgroup of patients who have been shown to have lower drug survival than biologic‐naïve users.^38^, ^39^ Notably, drug survival for all three groups was similar for approximately 6 months, at which point the survival curves separated and ixekizumab performed better out to 24 months of follow‐up.

The results from the current study, which found ixekizumab initiators experienced longer drug survival compared with TNFi and non‐ixekizumab IL‐17 initiators, are also consistent with studies that have investigated drug survival of ixekizumab in a real‐world setting. A small, retrospective, clinic‐based study conducted between 22 March 2016, (FDA approval date for the use of ixekizumab for plaque psoriasis) and 25 November 2018, found that 14 of 22 patients (64%) were still taking ixekizumab at the end of the study period and had been taking it for an average of 379 days.^40^ In a cohort of patients from the Danish national registry for PsO patients on biologics (DERMBIO), researchers examined 368 secukinumab and 62 ixekizumab patients and found that drug survival was higher for ixekizumab patients, whether biologic‐naïve or biologic‐experienced.^33^ Although both studies found longer drug survival in ixekizumab treated patients, both are hindered by small ixekizumab sample sizes.

Despite the efficacy and safety profile described for the newer generation of biologics in the treatment of psoriatic diseases, Murage et al^41^ found that adherence and persistence rates ranged from 50% to 60% for PsO, rheumatoid arthritis, and psoriatic arthritis. Long‐term safety, higher comorbidities, administrative challenges, and costs were found to be factors potentially responsible for the low biologic adherence and persistence rates. Dissatisfaction with current treatment options, moreover, may reflect a patient preference to continue, discontinue, or not to initiate a drug. A sound patient‐physician dialogue can assist in the management of patient expectations and helps the clinician identifying the appropriate biologic, increasing the chances for long term success and best patient outcomes. Psoriasis treatments are quickly evolving with the availability of newer biologics. For a more comprehensive understanding of a drug profile, in addition to clinical trial data, clinicians should consider real‐world evidence data. Drug survival and cost‐effectiveness are valuable measures to assess the drug performance in real patients and settings, and these should become important indicators when choosing the appropriate treatment for the best patient outcomes.

Our study is not without limitations. The data source is a North American Registry, and the results may not be generalizable to other regions. The data are subject to limitations inherent in all observational studies, such as the lack of randomization to patients for different biologics and the unknown patient factors that may be associated with access to care. Estimation of drug survival between treatments should also be cautiously interpreted when using nonrandomized data. Nevertheless, our study has important strengths. Data for the current study were collected across the United States and Canada from both academic and private practice dermatologists, and these patients are more likely to reflect the typical real‐world patient population than those in clinical trials. The duration and 2‐year follow up of the current study were reasonable and clinically meaningful. We were able to adjust for several potential confounders, including baseline disease severity, clinical outcomes, and treatment patterns, which are not captured in claims databases.

## 5 CONCLUSIONS

The current study from a large cohort of North American PsO patients demonstrates that over 2 years ixekizumab has a better drug survival compared with TNFi and non‐ixekizumab IL‐17i. The findings were consistent in both biologic‐naïve and biologic‐experienced patients. The current study data offer valuable evidence regarding drug performance and real‐life differences, which may be taken into clinicians' consideration when choosing between biologics.

## CONFLICTS OF INTEREST

Benjamin Lockshin, Grant/Research Support: AbbVie, Regeneron, Lilly, Franklin, DermTech, Trevi, Dermira, Celgene, Novartis, LEO, Strata; Consultant: AbbVie, DermTech; Speakers Bureau: AbbVie, Lilly, Novartis, Regeneron, Sanofi; Angel Cronin, Employee of Corrona LLC; Ryan W. Harrison, Employee of Corrona LLC; Robert R. McLean, Employee of Corrona LLC; Laura Anatale‐Tardiff, Employee of Corrona LLC; Russel Burge, Employee/Stock, Eli Lilly and Company; Baojin Zhu, Employee/Stock, Eli Lilly and Company; William N. Malatestinic, Employee/Stock, Eli Lilly and Company; Bilal Atiya, Employee/Stock, Eli Lilly and Company; Mwangi J. Murage, Employee/Stock, Eli Lilly and Company; Gaia Gallo, Employee/Stock, Eli Lilly and Company; Bruce Strober, Investigator: AbbVie, Dermavant, Corrona, Dermira; Speaker for AbbVie, Lilly, Janssen, Ortho Dermatologics; Consultant (Honoraria): AbbVie, Almirall, Amgen, Arena, Aristea Therapeutics, Boehringer Ingelheim, Bristol Myers Squibb, Celgene, Dermavant, Dermira, Janssen, LEO Pharma, Eli Lilly, Kyowa Hakko Kirin, Meiji Seika Pharma, Novartis, Pfizer, GlaxoSmithKline, UCB, Sun Pharma, Ortho Dermatologics, Regeneron, Sanofi‐Genzyme; Scientific Director (consulting fee): Corrona Psoriasis Registry; Abby Van Voorhees, Advisory Board: Celgene; Consultant: Celgene, DermTech, Lilly, ICOS, Novartis, UCB, WebMD; Grants/Research Support: AbbVie, Celgene, Lilly, ICOS; Other: Merck.

## Supporting information


**TABLE S1** Adjusted model 1 (prespecified covariates): hazard ratios from multivariable Cox proportional hazards regression
**TABLE S2** Adjusted model 2 (prespecified covariates plus consideration of characteristics with standardized differences >0.10): hazard ratios from multivariable Cox proportional hazards regression

## Data Availability

The Corrona dataset is based on a large US North American multicenter study adhering to a number of institutional review boards, with complex logistics. Patients did not provide consent to raw data sharing during the data collection for this purpose, and the Corrona data sharing policies do not permit raw data sharing for this purpose. An aggregated limited dataset from the current analyses is available to qualified investigators with an approved protocol. Data requests may be sent to Corrona, represented by Dr. Jeffrey D. Greenberg MD MPH, NYU School of Medicine, New York, NY, e‐mail jgreenberg@corrona.org.
