# Supplementary material for: Drug survival of ixekizumab, TNF inhibitors, and other IL‐17 inhibitors in real‐world patients with psoriasis: The Corrona Psoriasis Registry
Source: Dermatol Ther. 2021 Feb 15;34(2):e14808. doi: 10.1111/dth.14808 (PMC8047872; doi:10.1111/dth.14808)
Supplement: Supplementary file 1 — TABLE S1 Adjusted model 1 (prespecified covariates): hazard ratios from multivariable Cox proportional hazards regression TABLE S2 Adjusted model 2 (prespecified covariates plus consideration of characteristics with standardized differences >0.10): hazard ratios from multivariable Cox proportional hazards regression [file DTH-34-e14808-s001.docx]

**Appendices**

Supplemental Table 1. Adjusted model 1 (pre-specified covariates): hazard ratios from multivariable Cox proportional hazards regression

|  | **Main analysis** | | | **Sensitivity analysis**  **(subgroup with BSA>3, PASI>3, and IGA>1 at index)** | | |
| --- | --- | --- | --- | --- | --- | --- |
|  | n=1,594 initiations  n=722 discontinuations | | | n=1,070 initiations  n=483 discontinuations | | |
|  | *Adjusted HR | 95% CI | P-value | *Adjusted HR | 95% CI | P-value |
| Drug group |  |  |  |  |  |  |
| ixekizumab vs. TNFi (ref) | 0.33 | 0.25, 0.43 | <0.001 | 0.31 | 0.23, 0.43 | <0.001 |
| -xekizumab vs. non-ixekizumab IL-17i (ref) | 0.68 | 0.54, 0.86 | 0.001 | 0.61 | 0.46, 0.80 | <0.001 |
| Age (per SD increase**) | 1.03 | 0.93, 1.14 | 0.59 | 1.08 | 0.97, 1.22 | 0.17 |
| Gender (female vs. male) | 1.89 | 1.54, 2.33 | <0.001 | 1.80 | 1.43, 2.26 | <0.001 |
| Weight (per SD increase**) | 1.13 | 1.02, 1.25 | 0.02 | 1.21 | 1.09, 1.35 | <0.001 |
| Dermatologist identified PsA (yes vs. no) | 1.33 | 1.08, 1.63 | 0.007 | 1.13 | 0.90, 1.42 | 0.29 |
| Duration of psoriatic disease |  |  |  |  |  |  |
| <5 years | Reference |  |  | Reference |  |  |
| 5 to <10 years | 0.48 | 0.34, 0.66 | <0.001 | 0.56 | 0.39, 0.82 | 0.003 |
| 10 to <15 years | 0.54 | 0.39, 0.74 | <0.001 | 0.67 | 0.46, 0.96 | 0.03 |
| 15 to <20 years | 0.63 | 0.44, 0.91 | 0.01 | 0.65 | 0.44, 0.96 | 0.03 |
| >20 years | 0.59 | 0.45, 0.77 | <0.001 | 0.58 | 0.43, 0.79 | <0.001 |
| History of prior biologics |  |  |  |  |  |  |
| Biologic naive | Reference |  |  | Reference |  |  |
| 1 prior biologic | 1.35 | 1.05, 1.75 | 0.02 | 1.42 | 1.06, 1.92 | 0.02 |
| >2 prior biologics | 1.53 | 1.17, 1.98 | 0.002 | 1.68 | 1.24, 2.26 | <0.001 |

Hazard ratios (HR) less than 1 correspond to prolonged drug survival; BSA, Body surface area; PASI, Psoriasis Area and Severity Index; IGA, Investigator Global Assessment; CI, Confidence Interval; SD, Standard Deviation; *Adjusted for variables listed in the table; **The SD for age is 14 years and the SD for weight is 25kg (or 54lb)

Supplemental Table 2. Adjusted model 2 (pre-specified covariates plus consideration of characteristics with standardized differences >0.10): hazard ratios from multivariable Cox proportional hazards regression

|  | **Main analysis** | | | **Sensitivity analysis**  **(subgroup with BSA>3, PASI>3, and IGA>1 at index)** | | |
| --- | --- | --- | --- | --- | --- | --- |
|  | n=1,566 initiations  n=710 discontinuations | | | n=1,051 initiations  n=472 discontinuations | | |
|  | *Adjusted HR | 95% CI | P value | *Adjusted HR | 95% CI | P-value |
| Drug group |  |  |  |  |  |  |
| ixekizumab vs. TNFi (ref) | 0.36 | 0.27, 0.47 | <0.001 | 0.35 | 0.26, 0.47 | <0.001 |
| ixekizumab v.s non-ixekizumab IL-17i (ref) | 0.69 | 0.55, 0.87 | 0.002 | 0.62 | 0.48, 0.82 | <0.001 |
| Age (per SD increase**) | 1.00 | 0.88, 1.13 | 0.96 | 1.06 | 0.92, 1.22 | 0.43 |
| Gender (female vs. male) | 1.60 | 1.29, 1.99 | <0.001 | 1.57 | 1.23, 1.99 | <0.001 |
| Weight (per SD increase**) | 1.09 | 0.97, 1.21 | 0.13 | 1.17 | 1.04, 1.31 | 0.009 |
| Dermatologist identified PsA (yes vs. no) | 1.17 | 0.94, 1.44 | 0.15 | 1.00 | 0.79, 1.26 | 0.98 |
| Duration of psoriatic disease |  |  |  |  |  |  |
| <5 years | Reference |  |  | Reference |  |  |
| 5 to <10 years | 0.52 | 0.37, 0.71 | <0.001 | 0.63 | 0.44, 0.91 | 0.01 |
| 10 to <15 years | 0.61 | 0.44, 0.85 | 0.003 | 0.77 | 0.54, 1.10 | 0.15 |
| 15 to <20 years | 0.69 | 0.49, 0.99 | 0.04 | 0.74 | 0.51, 1.09 | 0.13 |
| 20 years | 0.68 | 0.52, 0.89 | 0.005 | 0.65 | 0.48, 0.88 | 0.006 |
| His>tory of prior biologics |  |  |  |  |  |  |
| Biologic naive | Reference |  |  | Reference |  |  |
| 1 prior biologic | 1.44 | 1.11, 1.87 | 0.007 | 1.39 | 1.03, 1.86 | 0.03 |
| >2 prior biologics | 1.54 | 1.18, 2.02 | 0.001 | 1.63 | 1.21, 2.19 | 0.001 |
| Race |  |  |  |  |  |  |
| White | Reference |  |  | Reference |  |  |
| African-American | 0.92 | 0.56, 1.54 | 0.76 | 0.95 | 0.55, 1.65 | 0.85 |
| Asian | 0.66 | 0.44, 0.99 | 0.04 | 0.70 | 0.46, 1.09 | 0.11 |
| Other | 0.74 | 0.52, 1.06 | 0.10 | 0.76 | 0.52, 1.13 | 0.17 |
| Education |  |  |  |  |  |  |
| 12th grade or less | Reference |  |  | Reference |  |  |
| High school graduate/GED | 0.89 | 0.60, 1.32 | 0.56 | 0.93 | 0.61, 1.43 | 0.74 |
| Some college/associates degree | 0.94 | 0.63, 1.38 | 0.74 | 0.97 | 0.63, 1.49 | 0.90 |
| College graduate or higher | 0.86 | 0.58, 1.27 | 0.46 | 0.99 | 0.64, 1.52 | 0.96 |
| Work Status |  |  |  |  |  |  |
| Full-time | Reference |  |  | Reference |  |  |
| Part-time | 1.38 | 0.96, 1.98 | 0.08 | 1.44 | 0.96, 2.16 | 0.08 |
| Work at home | 1.24 | 0.86, 1.81 | 0.25 | 1.35 | 0.89, 2.03 | 0.16 |
| Student | 1.54 | 0.77, 3.08 | 0.22 | 1.96 | 0.93, 4.12 | 0.08 |
| Disabled | 1.34 | 0.95, 1.90 | 0.10 | 1.72 | 1.17, 2.53 | 0.005 |
| Retired | 1.13 | 0.81, 1.58 | 0.46 | 1.12 | 0.77, 1.63 | 0.55 |
| Geographic region |  |  |  |  |  |  |
| US Northeast | Reference |  |  | Reference |  |  |
| US Midwest | 0.58 | 0.42, 0.81 | 0.002 | 0.69 | 0.47, 1.00 | 0.05 |
| US South | 0.95 | 0.73, 1.23 | 0.68 | 0.96 | 0.72, 1.27 | 0.77 |
| US West | 0.92 | 0.63, 1.34 | 0.66 | 0.89 | 0.59, 1.34 | 0.57 |
| Canada | 0.96 | 0.52, 1.78 | 0.90 | 0.88 | 0.46, 1.69 | 0.70 |
| Smoking history |  |  |  |  |  |  |
| Never smoked | Reference |  |  | Reference |  |  |
| Former smoker | 1.17 | 0.93, 1.47 | 0.19 | 1.28 | 0.99, 1.65 | 0.06 |
| Current smoker | 1.49 | 1.13, 1.96 | 0.004 | 1.80 | 1.33, 2.43 | <0.001 |
| History of Crohn's disease, Ulcerative Colitis, Indeterminate IBD, or other GI disorders (yes vs. no) | 0.96 | 0.73, 1.26 | 0.76 | 0.91 | 0.67, 1.25 | 0.57 |
| History of infections (yes vs. no) | 1.21 | 0.99, 1.48 | 0.06 | 1.25 | 1.00, 1.57 | 0.05 |
| Erythrodermic morphology (yes vs no) | 0.92 | 0.50, 1.68 | 0.79 | 1.02 | 0.56, 1.84 | 0.96 |
| Inverse/intertriginous morphology (yes vs. no) | 0.90 | 0.62, 1.30 | 0.57 | 1.10 | 0.75, 1.63 | 0.62 |
| Nail morphology (yes vs. no) | 0.90 | 0.67, 1.19 | 0.45 | 0.85 | 0.62, 1.16 | 0.30 |
| PASI (>12 vs. <12) | 0.78 | 0.60, 1.02 | 0.07 | 0.85 | 0.65, 1.10 | 0.21 |
| IGA (2, 3, 4 vs. 0, 1) | 1.37 | 0.94, 2.01 | 0.10 | -- | -- | -- |
| Concomitant topical therapy (yes vs. no) | 1.05 | 0.87, 1.28 | 0.60 | 1.04 | 0.84, 1.29 | 0.71 |
| Patient overall itch/pruritis VAS (per SD increase**) | 1.09 | 0.99, 1.22 | 0.09 | 1.07 | 0.95, 1.21 | 0.27 |
| Patient health state today (EQ-VAS, per SD increase**) | 0.96 | 0.87, 1.06 | 0.43 | 1.04 | 0.93, 1.16 | 0.50 |

Hazard ratios (HR) less than 1 correspond to prolonged drug survival; BSA, Body surface area; PASI, Psoriasis Area and Severity Index; IGA, Investigator Global Assessment; CI, Confidence Interval; SD, Standard Deviation; *Adjusted for variables listed in the table; **The SD for age is 14 years, the SD for weight is 25kg (or 54lb), the SD increase for patient overall itch/pruritis is VAS (33.3), and for EQ-VAS (21.3)
